# Supplementary material for: A strategy for tough and fatigue-resistant hydrogels via loose cross-linking and dense dehydration-induced entanglements
Source: Nat Commun. 2024 Jul 13;15:5896. doi: 10.1038/s41467-024-50364-3 (PMC11246433; doi:10.1038/s41467-024-50364-3)
Supplement: Supplementary file 1 — Supplementary Information [file 41467_2024_50364_MOESM1_ESM.pdf]

Supplementary Information

A strategy for tough and fatigue-resistant hydrogels via loose cross-linking and dense dehydration-induced entanglements

Danming Zhong[1,4], Zhicheng Wang[1,4], Junwei Xu[1], Junjie Liu[2], Rui Xiao[1], Shaoxing Qu[1,3]*, Wei Yang[1]

[1]State Key Laboratory of Fluid Power & Mechatronic System, Key Laboratory of Soft Machines and Smart Devices of Zhejiang Province, Center for X-Mechanics, Department of Engineering Mechanics, Zhejiang University, Hangzhou, 310027, China.
[2]Applied Mechanics and Structure Safety Key Laboratory of Sichuan Province, School of Mechanics and Aerospace Engineering, Southwest Jiaotong University, Chengdu, 611756, China.
[3]Eye Center, The Second Affiliated Hospital, School of Medicine, Zhejiang University, Hangzhou, 310009, China.
[4]These authors contributed equally: Danming Zhong, Zhicheng Wang.

*Corresponding author: Shaoxing Qu, squ@zju.edu.cn

Supplementary Notes

Supplementary Note 1. A hyperelastic model considering cross-linking and entanglements.

Microscopically, a hydrogel consists of cross-linked polymer chains, interchain entanglements, and free chains. The free chains will not be discussed here since they are irrelevant to the quasi-static stress-stretch ratio (s-s) curves tested in this paper. The hyperelastic strain energy function comprises contributions from both the cross-linked network and the entanglements. We developed a visco-hyperelastic model, and the rationality of this model was validated by experiments of medium cross-linked hydrogels (*1*). We employ this model to describe the hyperelastic behaviors of hydrogels with various degrees of cross-linking and dehydration. The model is briefly reviewed here.

The Langevin probability function was adopted to quantify the distribution of the end-to-end distance of a cross-linked chain (*1*). The interchain entanglements were characterized using a tube model (*2*). Accordingly, the strain energy density function is expressed as

$$W_{hyper} = \underbrace{G_c N \ln\left(\frac{3N+\frac{1}{2}I_1}{3N-I_1}\right)}_{W^c_{hyper}} + \underbrace{G_e \sum_{i=1,2,3} \frac{1}{\lambda_i}}_{W^e_{hyper}}, \quad (1)$$

with three model parameters: the cross-linked shear modulus G_c for the cross-linked network, the entangled shear modulus G_e for the entangled chains, and the number of Kuhn segments N in a single chain. λ_i is the principal stretch ratio along the ith principal direction. Assuming the hydrogel is incompressible, the elastic modulus is three times the shear modulus, i.e., $E_c=3G_c$ and $E_e=3G_e$. The Cauchy stress is calculated by the derivative of the free energy density (Eq.(1)) with respect to the deformation gradient tensor **F**:

$$\boldsymbol{\sigma}_{hyper} = \underbrace{\frac{1}{J}\frac{\partial W^c_{hyper}}{\partial \mathbf{F}}\mathbf{F}^T}_{\boldsymbol{\sigma}^c_{hyper}} + \underbrace{\frac{1}{J}\frac{\partial W^e_{hyper}}{\partial \mathbf{F}}\mathbf{F}^T}_{\boldsymbol{\sigma}^e_{hyper}} + p\mathbf{I}, \quad (2)$$

where J denotes the determinant of **F** and we have $J=1$ due to the incompressibility. The variable p is the hydrostatic pressure to reinforce volumetric incompressibility with **I** as a second rank identity tensor. Eq.(2) describes the constitutive relationship of a hydrogel. Taking uniaxial tension as an example, the nominal stress is expressed as

$$s_1 = G_c \frac{\lambda_1 - \lambda_1^{-2}}{(1-(\lambda_1^2+2\lambda_1^{-1})/3/N)(1+0.5(\lambda_1^2+2\lambda_1^{-1})/3/N)} + G_e(\lambda_1^{-1/2} - \lambda_1^{-2}), \quad (3)$$

with λ_1 being the stretch ratio along the stretch direction.

Once the hydrogel is dehydrated, both the cross-linked modulus G_c and the

entangled modulus G_e increase with the polymer content ϕ_p. The cross-linked modulus satisfies (*1*):

$$G_c(\phi_p) = G_c(\phi_{p0})(\frac{\phi_p}{\phi_{p0}})^{\frac{9\nu-4}{3(3\nu-1)}} \tag{4}$$

with $G_c(\phi_{p0})$ being the cross-linked shear modulus at the initial polymer content ϕ_{p0}, and ν being a scaling exponent. The entangled modulus satisfies another scaling law (*1*):

$$G_e(\phi_p) = \begin{cases} \frac{G_e(\phi_e)}{\phi_e}\phi_p, & \phi_p^* < \phi_p \le \phi_e, \\ G_e(\phi_e)(\frac{\phi_p}{\phi_e})^{3\nu/(3\nu-1)}, & \phi_e < \phi_p < \phi_p^{**}. \end{cases} \tag{5}$$

where ϕ_e is the critical polymer content which corresponds to the boundary of unentangled and entangled regimes, and $G_e(\phi_e)$ is the entangled shear modulus when $\phi_p = \phi_e$. The polymer contents ϕ_p^* and ϕ_p^{**} indicate the boundaries between the dilute solution, semi-dilute solution, and concentrated solution (*3*). For the precursor solution to form a solid hydrogel, it must satisfy $\phi_p > \phi_p^*$.

The experimental quasi-static s-s curves were compared with the theoretical curves predicted by Eqs. (3)-(5).

Supplementary Note 2. Determination of the critical polymer content ϕ_e for LC/MC/HC hydrogels.

Consider an ideal cross-linked hydrogel in which no entanglements are formed during dehydration. For this hydrogel, swelling or dehydration leads to volume expansion or shrinkage, respectively, causing changes in chain density while keeping the number of polymer chains within the cross-section constant. The orientation distribution of polymer chains in the hydrogel remains unchanged throughout the swelling or dehydration process. It is further assumed that only the polymer chains in the hydrogel carry the load and the water molecules do not. As a result, in the small deformation region of the hydrogel, where the deformation of polymer chains is far from their stretch limits, the force-strain curve will remain relatively unchanged after swelling or dehydration. On the contrary, if entanglements are formed after dehydration, the force-strain curve should rise considerably due to the additional contribution from entanglements. Based on the above analysis, we determine the critical polymer content ϕ_e of entanglements by comparing the overlap of force-strain curves. For the LC hydrogels, the force-strain curves rise significantly as the water content decreases from 93% to 78% (Supplementary Fig. 1A), indicating that the critical polymer content ϕ_e of LC hydrogels should be less than 7%. For MC hydrogels, the force-strain curves for hydrogels with water contents of ϕ_w =93%, 90%, and 87% basically coincide (Supplementary Fig. 1B), and they are lower than that of the water content of ϕ_w=78%.

Accordingly, the critical polymer content ϕ_e of MC hydrogels should fall within the range of 13% to 22%. In a similar manner, the ϕ_e of HC hydrogels is estimated to be in the range of 30% to 38% (Supplementary Fig. 1C).

Based on the acquired distribution range of the critical polymer contents, we compare the experimental quasi-static s-s curves with the hyperelastic model. The experimental s-s curves within the range of λ=1.0 to λ=3.0 (i.e., the regions of small and medium deformation) are adopted. As previously mentioned, both the cross-linked network and the interchain entanglements contribute to the elastic modulus, thus the s-s curves in the small deformation region contain information about cross-linking and entanglement. As the stretch ratio further increases, a strain-softening phenomenon appears due to the removal of entanglements. Therefore, the experimental data we selected contain a wealth of information about the microstructure of hydrogels. Supplementary Fig. 2(A-C) shows the comparison of s-s curves for LC/MC/HC hydrogels with the corresponding theoretical curves. The fitted model parameters are listed in Supplementary Table 1. For hydrogels with various degrees of cross-linking and dehydration, the experimental curves agree with theoretical curves well.

Supplementary Figures

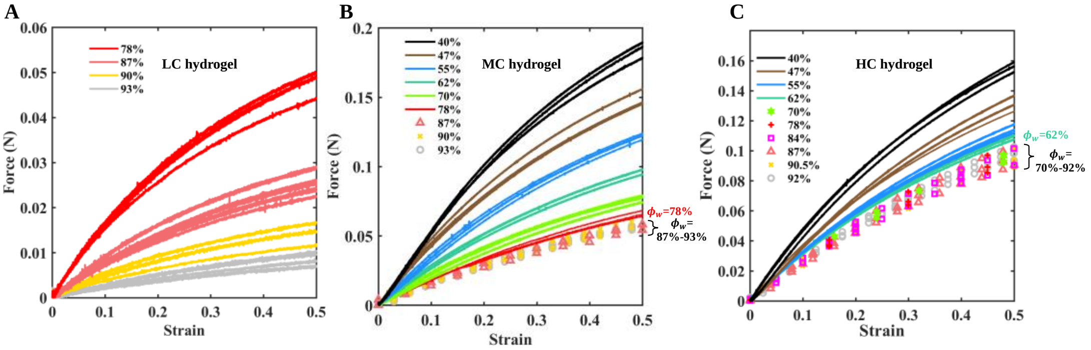

Supplementary Figure 1. Force-strain curves for (A) LC hydrogels, (B) MC hydrogels, and (C) HC hydrogels with various water contents. To better display the overlapping force-strain curves under different water contents, these curves are represented by data points. The critical polymer content ϕ_e is determined by assessing whether the force-strain curves overlap each other. The ϕ_e value of LC hydrogel is less than 7%. The ϕ_e values of MC hydrogel and HC hydrogel fall within the range of 13% to 22% and 30% to 38%, respectively.

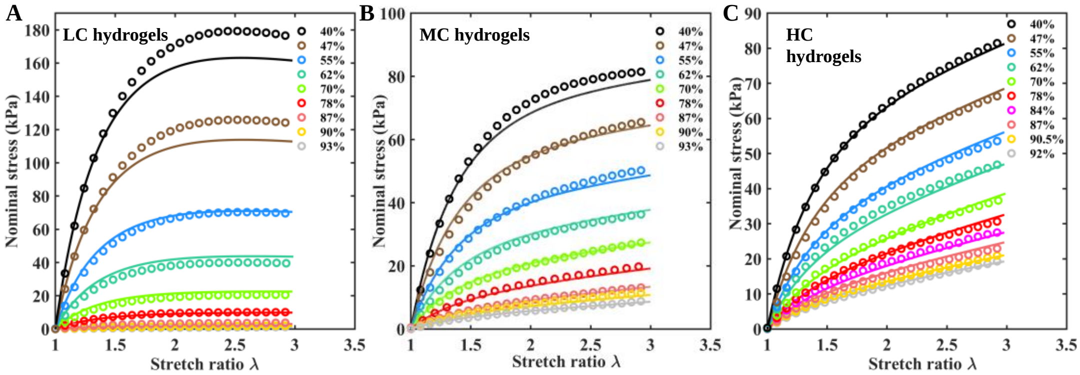

Supplementary Figure 2. Comparison of experimental s-s curves with theoretical curves obtained from a hyperelastic model: (A) LC hydrogels, (B) MC hydrogels, and (C) HC hydrogels. The theoretical curves are plotted using model parameters listed in Supplementary Table 1.

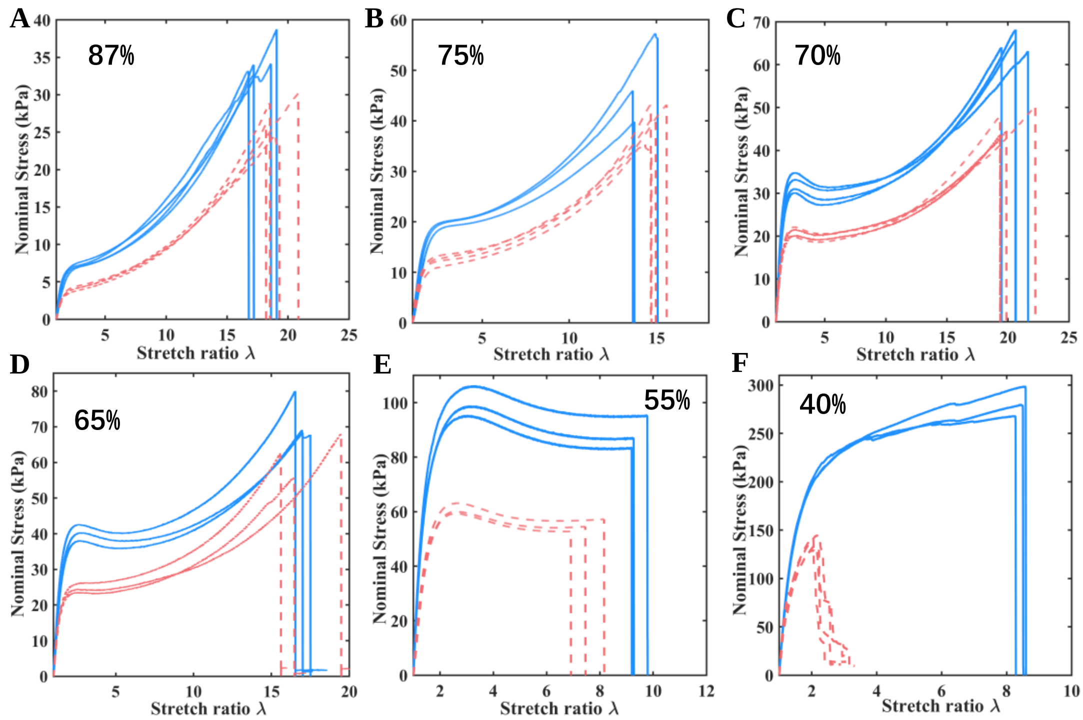

Supplementary Figure 3. S-s curves of uncut samples (blue solid lines) and precut samples (pink dotted lines) of LC hydrogels by pure shear tests. (A) ϕ_w=87%, (B) ϕ_w=75%, (C) ϕ_w=70%, (D) ϕ_w=65%, (E) ϕ_w=55%, (F) ϕ_w=40%. The samples have a width of 50 mm, a height of 10 mm, a thickness of about 2 mm, and a 20 mm-long crack for precut samples. The samples were stretched at a strain rate of $\dot{\varepsilon}$=0.1 s^{-1}.

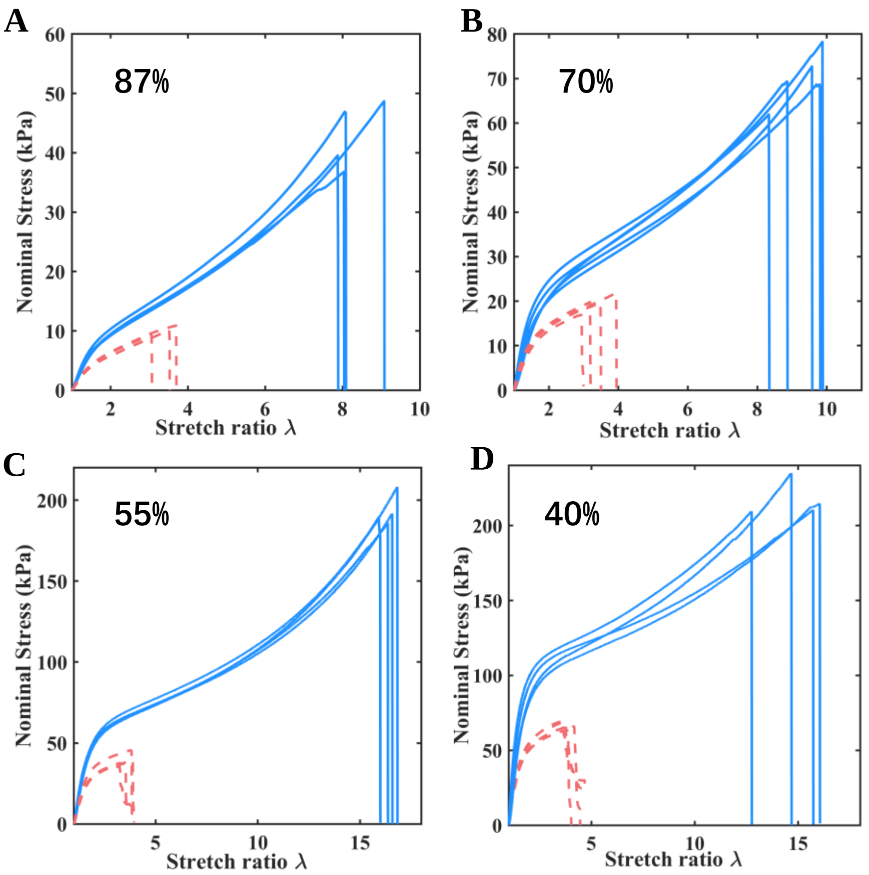

Supplementary Figure 4. S-s curves of uncut samples (blue solid lines) and precut samples (pink dotted lines) of MC hydrogels by pure shear tests. (A) ϕ_w=87%, (B) ϕ_w=70%, (C) ϕ_w=55%, (D) ϕ_w=40%. The samples have a width of 50 mm, a height of 10 mm, a thickness of about 2 mm, and a 20 mm-long crack for precut samples. The samples were stretched at a strain rate of $\dot{\varepsilon}$=0.1 s^{-1}.

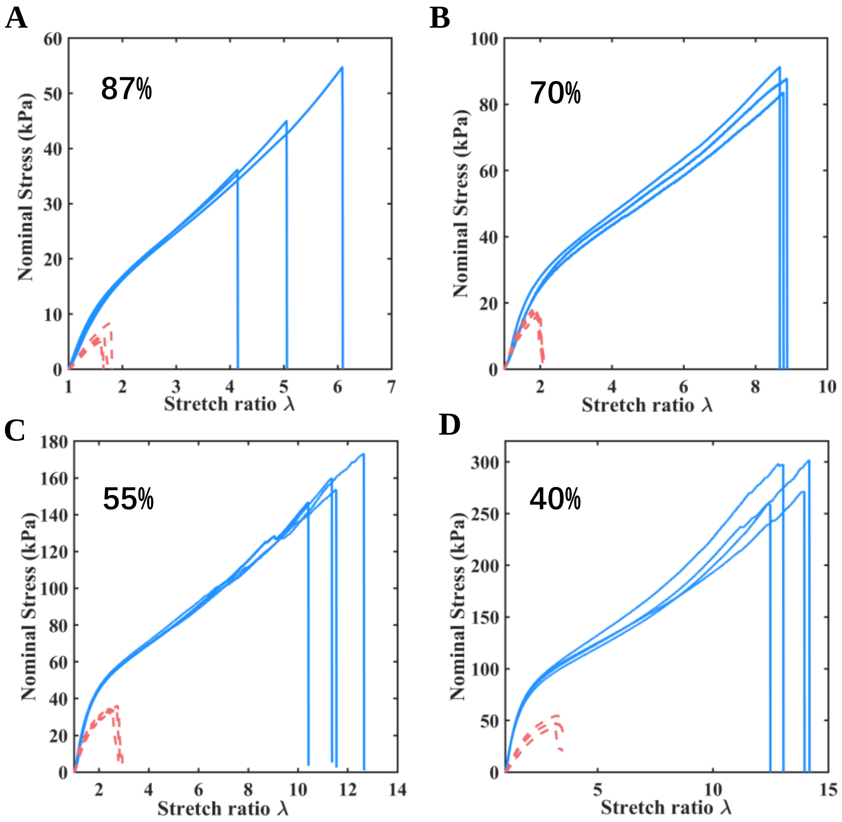

Supplementary Figure 5. S-s curves of uncut samples (blue solid lines) and precut samples (pink dotted lines) of HC hydrogels by pure shear tests. (A) ϕ_w=87%, (B) ϕ_w=70%, (C) ϕ_w=55%, (D) ϕ_w=40%. The samples have a width of 50 mm, a height of 10 mm, a thickness of about 2 mm, and a 20 mm-long crack for precut samples. The samples were stretched at a strain rate of $\dot{\varepsilon}$=0.1 s^{-1}.

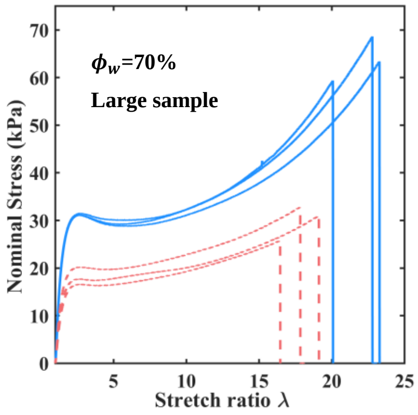

Supplementary Figure 6. S-s curves of uncut samples (blue solid lines) and precut samples (pink dotted lines) of LC hydrogels with ϕ_w=70%. Larger samples were adopted, with a width of 120 mm, a height of 40 mm, a thickness of about 3 mm, and a 48 mm-long crack for precut samples. The samples were stretched at a strain rate of $\dot{\varepsilon}$=0.1 s^{-1}. The fracture stretches of precut samples (λ_c=17.8) are obviously smaller than these of the uncut samples (λ_f=22.1). The fractocohesive length is 28.5 mm.

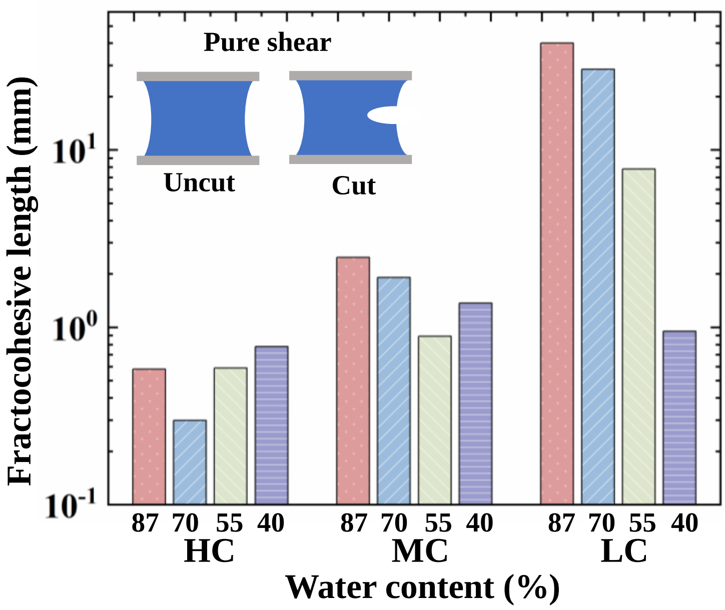

Supplementary Figure 7. Fractocohesive lengths of LC/MC/HC hydrogels with various water contents. The fractocohesive lengths of MC/HC hydrogels are small, within the range of 0.3 mm to 2.5 mm. The fractocohesive length of LC hydrogel with ϕ_w=87% should not be smaller than 40 mm, and it is taken as 40 mm here. The fractocohesive length of LC hydrogels decreases when dehydrated.

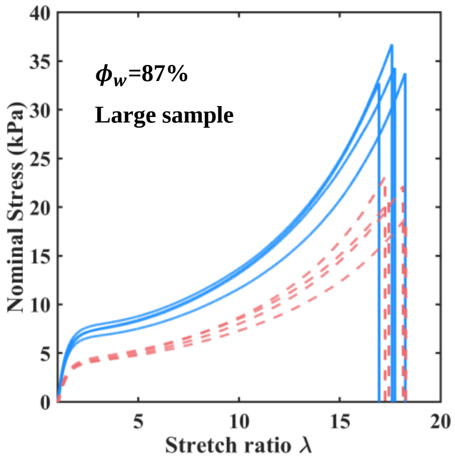

Supplementary Figure 8. S-s curves of uncut samples (blue solid lines) and precut samples (pink dotted lines) of LC hydrogels with ϕ_w=87%. Larger samples were adopted, with a width of 150 mm, a height of 40 mm, a thickness of about 3 mm, and a 60 mm-long crack for precut samples. The samples were stretched at a strain rate of $\dot{\varepsilon}$=0.1 s^{-1}. The fracture stretches of precut samples (λ_c=17.7) are close to these of the uncut samples (λ_f=17.6). Thus, the fractocohesive length should not be smaller than the height of the sample (40 mm).

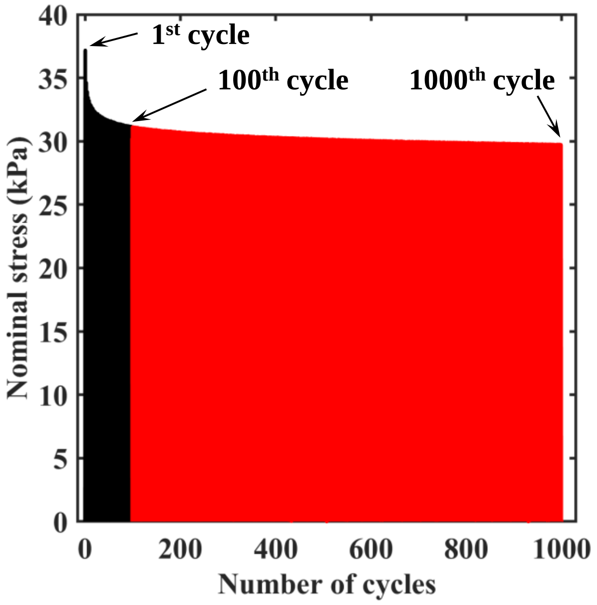

Supplementary Figure 9. Nominal stress-number of cycles curve of an uncut LC hydrogel with a water content of 70% during 1000 cycles. The stress attenuation between the 101st cycle and the 1000th cycle is negligible, measuring 3.9% of the peak stress of the 1st cycle. Therefore, the loading curve of the 100th cycle was taken to calculate the energy release rate.

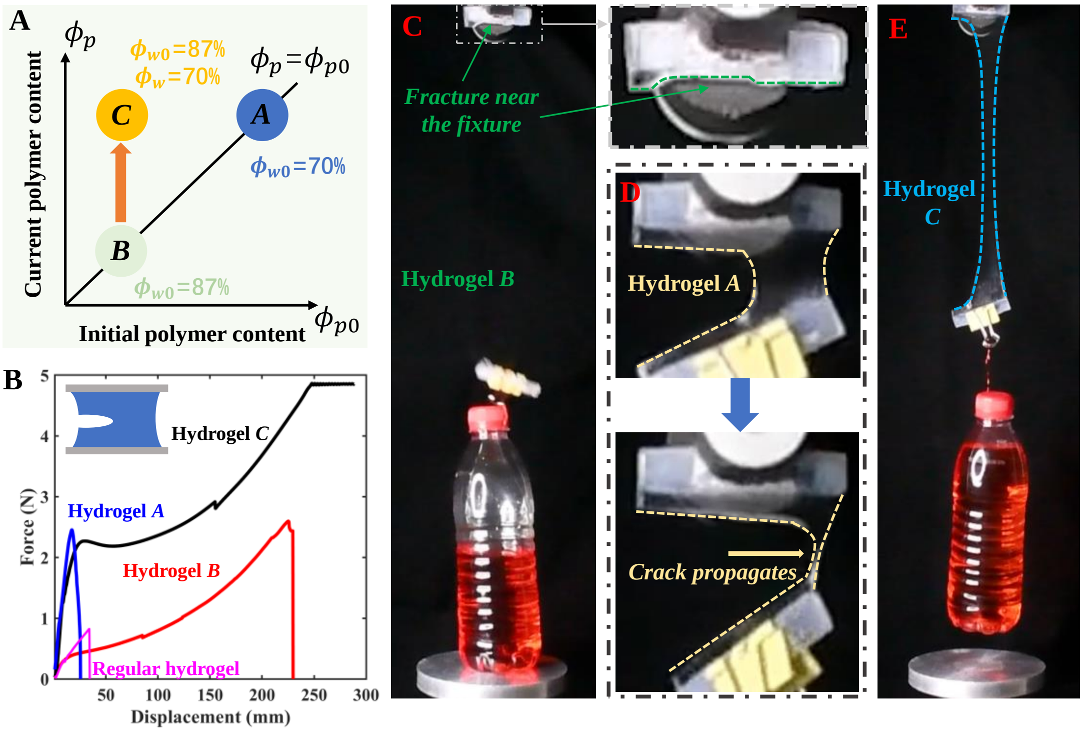

Supplementary Figure 10. Hydrogel samples (with a width of 50 mm, a height of 10 mm, a thickness of about 2 mm, and a 20 mm-long crack) were fabricated to lift a weight. (A) Three types of hydrogels with low cross-linking were prepared: hydrogel *A* with a water content of ϕ_{w0}=70%, hydrogel *B* with a water content of

ϕ_{w0}=87%, and hydrogel *C* prepared by the current strategy, with an initial water content of ϕ_{w0}=87% and a current water content of ϕ_w=70%. (B) The force-displacement curves for hydrogel *A*, *B*, *C*, and regular hydrogel. (C) Hydrogel *B* is not strong enough to lift a 300g weight. It fractures near the fixture. (D) Hydrogel *A* is not tough enough to lift a 300g weight. The precut crack propagates. (E) Hydrogel *C* is tough and strong enough to lift a 500g weight.

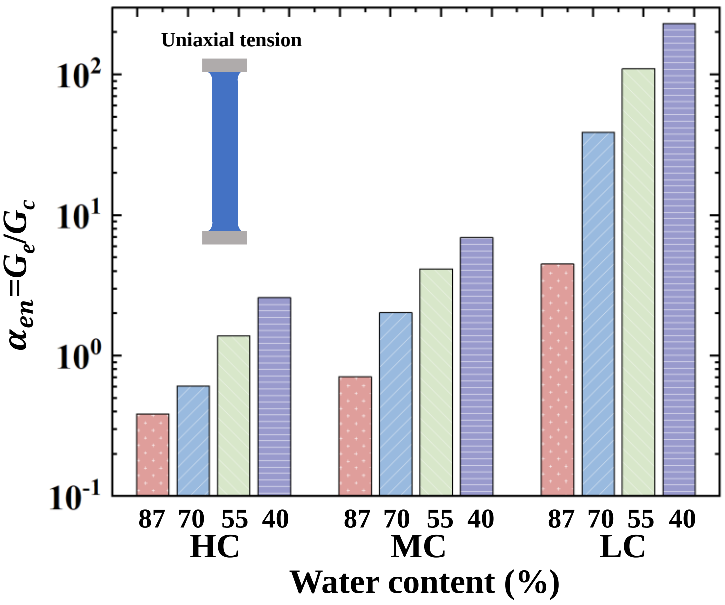

Supplementary Figure 11. The ratio of entangled modulus to cross-linked modulus (α_{en}) of LC/MC/HC hydrogels with various water contents. The α_{en} values were predicted using the hyperelastic model with parameters listed in Supplementary Table 1. The value of α_{en} will exceed 100 when a LC hydrogel is dehydrated to a water content of less than 55%, indicating the formation of a substantial number of dehydration-induced entanglements.

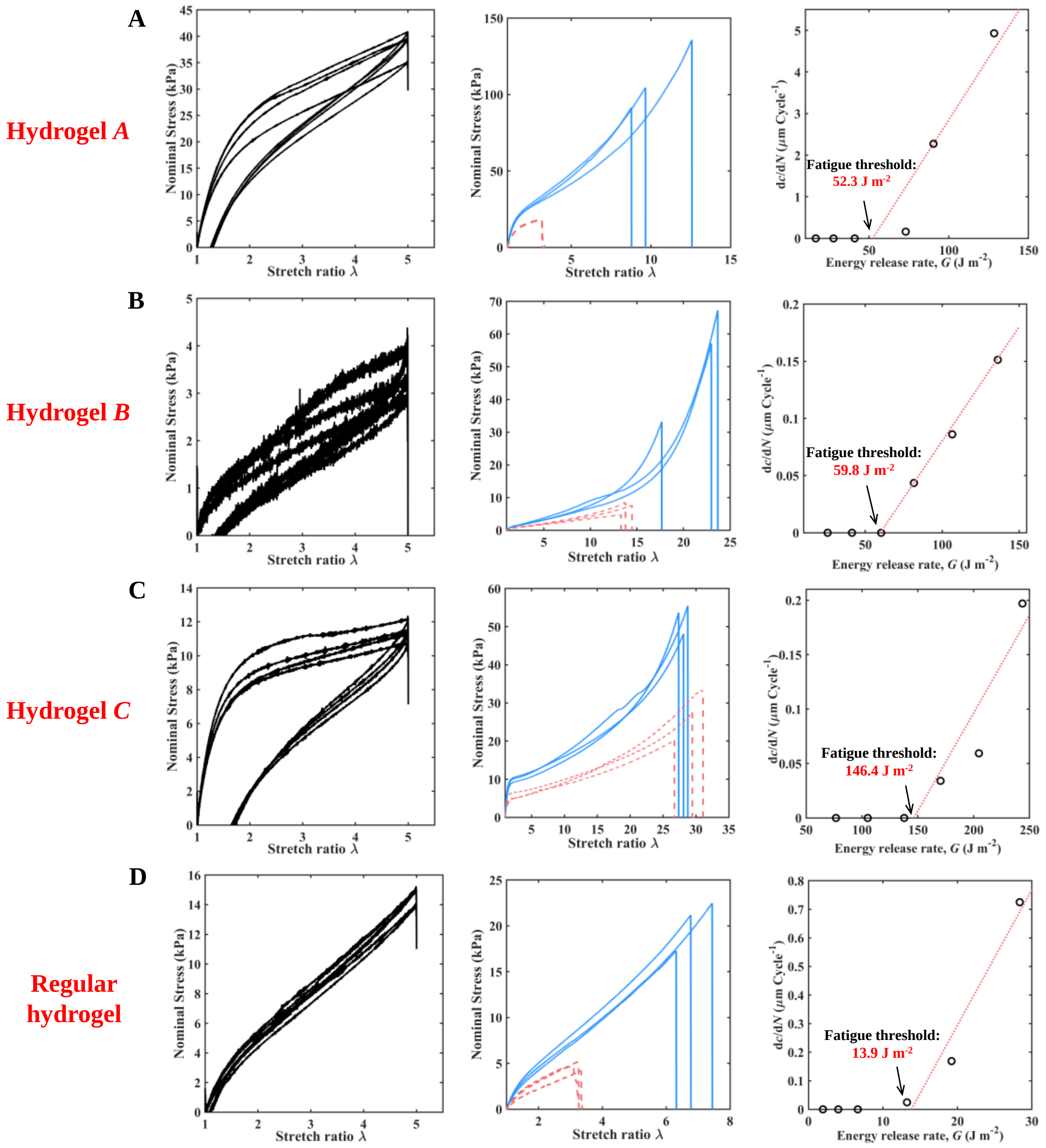

Supplementary Figure 12. Experimental curves of four types of PAAc hydrogels: (A) LC hydrogel *A* with ϕ_{w0}=70%. (B) LC hydrogel *B* with ϕ_{w0}=87%. (C) LC hydrogel *C* with an initial water content of ϕ_{w0}=87% and a current water content of ϕ_{w}=70%. (D) Regular MC hydrogel with ϕ_{w0}=87%. The first column is s-s curves of single-cycle tests at a strain rate of $\dot{\varepsilon}$=0.1 s^{-1}. The curves were adopted to calculate the elastic modulus. The second column is s-s curves of pure tests at a strain rate of $\dot{\varepsilon}$=0.1 s^{-1}. The curves were adopted to calculate the fracture toughness. The third column is the relationships between crack extension per cycle (d*c*/d*N*) and the energy release rate (*G*). The data were adopted to calculate the fatigue threshold.

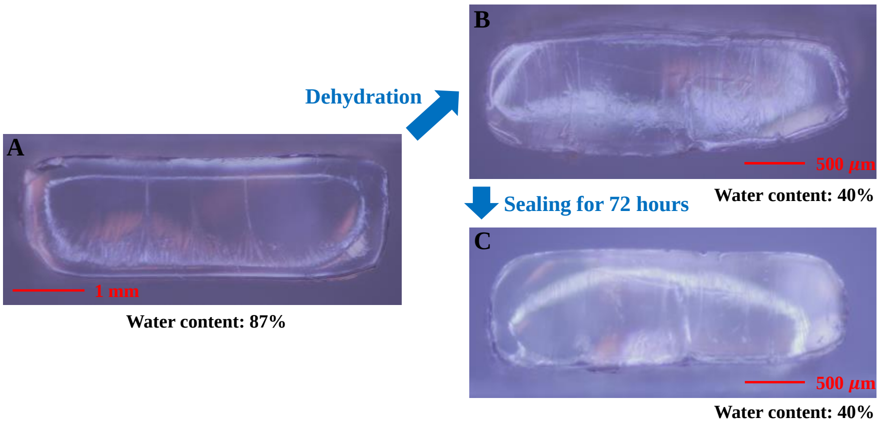

Supplementary Figure 13. Optical micrographs of cross-sections of hydrogel samples. (A) As-prepared hydrogel with a water content of 87%. The cross-section is a rectangle. The four vertices of the cross-section have a certain curvature, due to the limitations of the casting method. (B) Hydrogel that has just been dehydrated to a water content of 40%. Obviously, the cross-section looks like an ellipse, and the original two straight long sides become two curves, because the distribution of water content through the sample is quite nonuniform. (C) The dehydrated hydrogel that has been placed in a sealing bag for 72 hours. The cross-section turns back to a rectangle, and the two long sides become straight lines, indicating the uniformity of water content distribution.

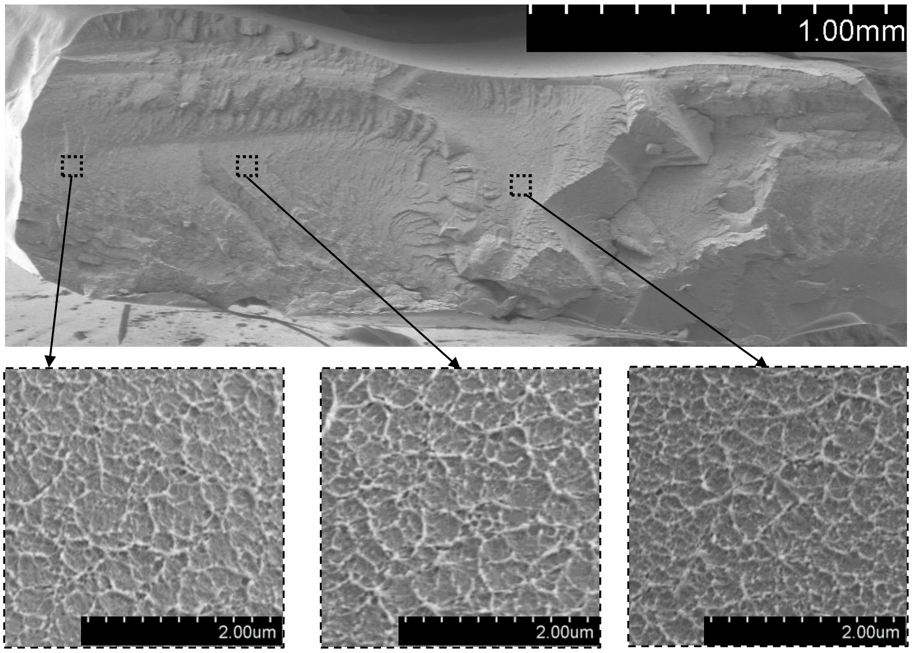

Supplementary Figure 14. SEM images of a dehydrated hydrogel sample with a water content of 40%. The uniformity of the polymer network structure at three various locations indicates that the distribution of water content through the dehydrated hydrogel should be uniform.

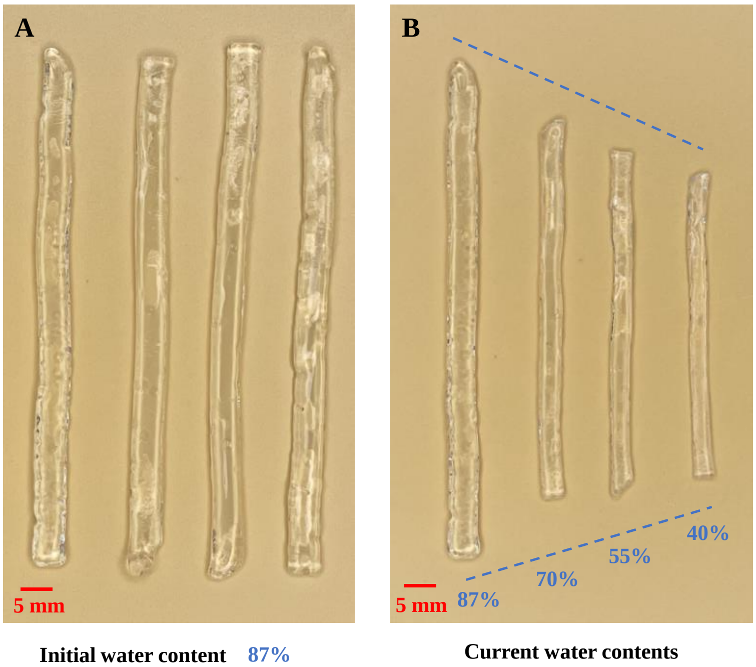

Supplementary Figure 15. Photos of PAAm hydrogel samples for uniaxial tension tests. (A) The as-prepared hydrogels with an initial water content of 87% have the same dimensions. (B) The dehydrated hydrogels with various current water contents. The lower the water content, the smaller the sample size.

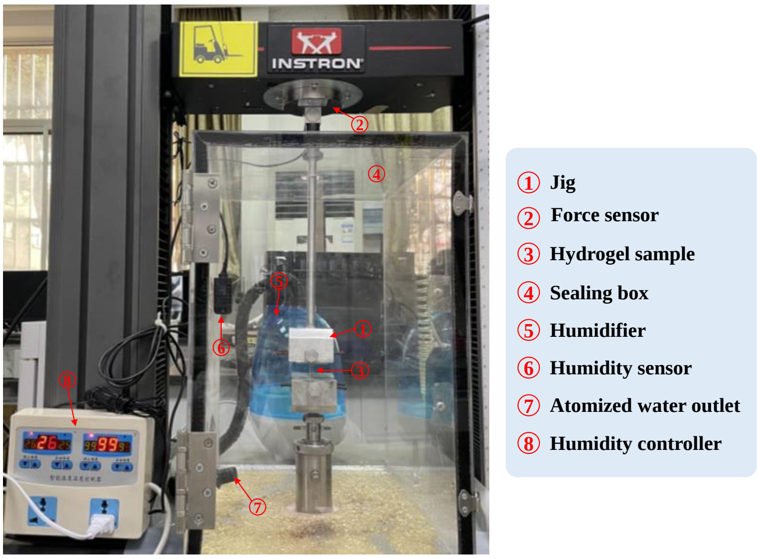

Supplementary Figure 16. Experimental setting of the quasi-static test of hydrogel samples. The platform consists of a mechanical test system (Instron 5965 with a force sensor of 10N), a humidity control system (a humidity sensor, a humidity controller, and a humidifier), and a sealing box.

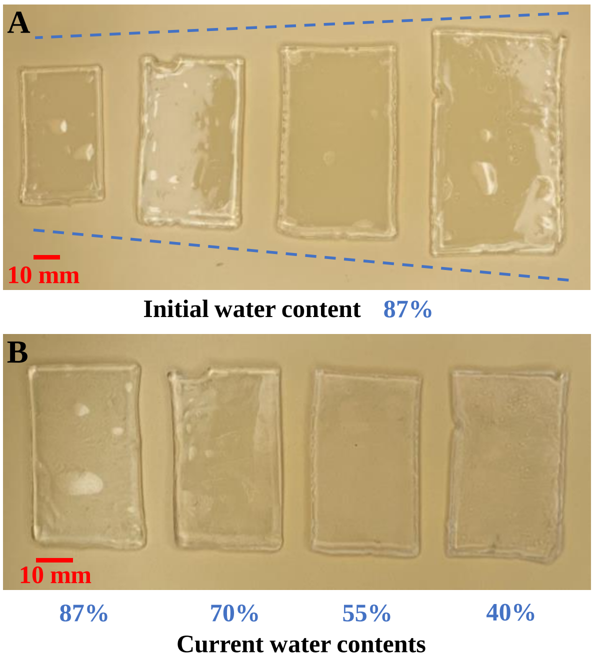

Supplementary Figure 17. Photos of PAAm hydrogel samples for pure shear tests. (A) The as-prepared hydrogels with an initial water content of 87% were designed with various initial sizes. The lower the targeted water content, the larger the size of the as-prepared hydrogel sample. (B) The dehydrated hydrogels with various current water contents have the same dimensions.

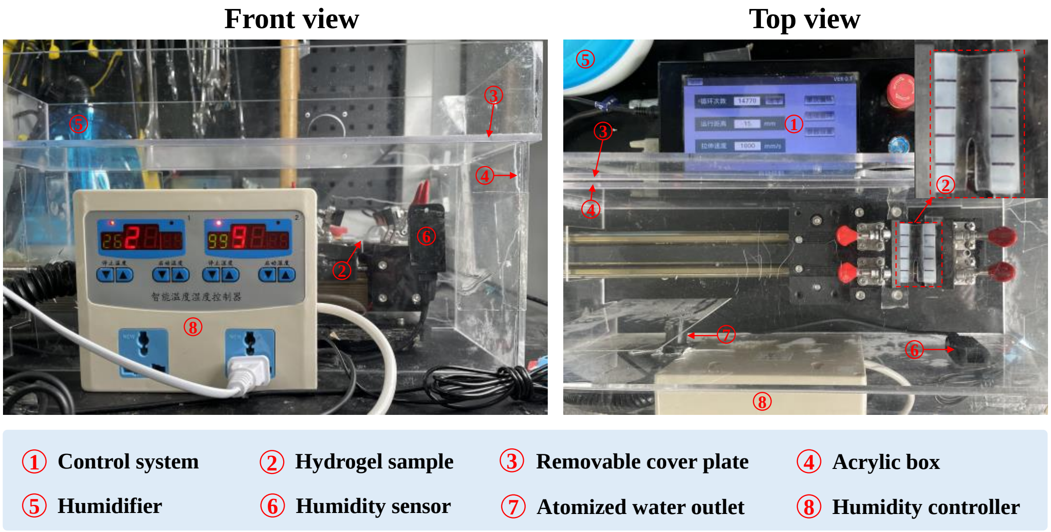

Supplementary Figure 18. Experimental setting of the fatigue test of hydrogel samples. The platform consists of a mechanical test system, a humidity control system (a humidity sensor, a humidity controller, and a humidifier), and a sealing box. The sealing box comprises an acrylic box (the lower part without a cover plate) and a removable acrylic cover plate (the upper part).

Supplementary Tables

Supplementary Table 1. Model parameters of LC/MC/HC PAAm hydrogels

	LC hydrogel	MC hydrogel	HC hydrogel
N	10000	1000	333
ϕ_e	<7.0%	18.0%	31.1%
$G_c(\phi_{p0})$ (kPa)	0.43	3.70	7.62
$G_e(\phi_{p0})$ (kPa)	1.93	2.64	2.96
ν	0.506	0.584	0.538

Supplementary Table 2. Modulus, fracture toughness, and fatigue threshold of four PAAm hydrogels

PAAm hydrogel	Hydrogel *A* (ϕ_{w0}=70%, LC)	Hydrogel *B* (ϕ_{w0}=87%, LC)	Hydrogel *C* (ϕ_{w0}=87%, ϕ_w=70%, LC)	Regular hydrogel (ϕ_{w0}=87%, MC)
Modulus (kPa)	104.9±9.0	15.2±0.6	86.9±7.9	22.4±2.1
Fracture toughness (J m^{-2})	448.1±12.5	4109.6±486.8	9782.5±429.78	391.2±20.4
Fatigue threshold (J m^{-2})	70.7	81.0	291.4	10.8

Supplementary Table 3. Modulus, fracture toughness, and fatigue threshold of four PAAc hydrogels

PAAc hydrogel	Hydrogel *A* (ϕ_{w0}=70%, LC)	Hydrogel *B* (ϕ_{w0}=87%, LC)	Hydrogel *C* (ϕ_{w0}=87%, ϕ_w=70%, LC)	Regular hydrogel (ϕ_{w0}=87%, MC)
Modulus (kPa)	51.6±4.3	4.1±0.9	26.3±3.2	8.7±1.6
Fracture toughness (J m^{-2})	477.1±27.7	926.5±103.4	6753.7±656.1	116.8±7.8
Fatigue threshold (J m^{-2})	52.3	59.8	146.4	13.9

Supplementary References:

1. Zhong, D., *et al*. A visco-hyperelastic model for hydrogels with tunable water content. *J. Mech. Phys. Solids* **173**, 105206 (2023).
2. Xiang, Y., *et al*. A physically based visco-hyperelastic constitutive model for soft materials. *J. Mech. Phys. Solids* **128**, 208-218 (2019).
3. Rubinstein, M. & Colby, R.H. Polymer physics. Oxford University Press (2003).
