# Supplementary material for: A strategy for tough and fatigue-resistant hydrogels via loose cross-linking and dense dehydration-induced entanglements
Source: Nat Commun. 2024 Jul 13;15:5896. doi: 10.1038/s41467-024-50364-3 (PMC11246433; doi:10.1038/s41467-024-50364-3)
Supplement: Supplementary file 3 — Description of Additional Supplementary Files [file 41467_2024_50364_MOESM3_ESM.pdf]

Description of Additional Supplementary Files

File Name: Supplementary Movie 1
Description: PAAm hydrogel B with a water content of ϕ_{w0}=87% and low cross-linking was prepared to lift a 300 g weight. Hydrogel B fractures near the fixture.

File Name: Supplementary Movie 2
Description: PAAm hydrogel A with a water content of ϕ_{w0}=70% and low cross-linking was prepared to lift a 300 g weight. The crack of hydrogel A propagates.

File Name: Supplementary Movie 3
Description: Regular PAAm hydrogel with a water content of ϕ_{w0}=87% and medium cross-linking was prepared to lift a 300 g weight. The crack of regular hydrogel propagates.

File Name: Supplementary Movie 4
Description: PAAm hydrogel C prepared by the current strategy, with an initial water content of ϕ_{w0}=87%, a current water content of ϕ_w =70% and low cross-linking was prepared to lift a 500 g weight. Hydrogel C lifted the weight successfully.
